# Does the Treatment of Pelvic Venous Insufficiency Really Not Influence Lower Limb Venous Disease?

**DOI:** 10.3390/diagnostics13152467

**Published:** 2023-07-25

**Authors:** Cezary Szary, Justyna Wilczko, Anna Bodziony, Krzysztof Celejewski, Siavash Swieczkowski-Feiz, Marcin Napierala, Dominika Plucinska, Michal Zawadzki, Jerzy Leszczynski, Tomasz Grzela

**Affiliations:** 1 Clinic of Phlebology, 02-034 Warsaw, Poland; 2 Medical University of Warsaw, 02-097 Warsaw, Poland; 3 Centre of Postgraduate Medical Education, 01-828 Warsaw, Poland

**Keywords:** descending treatment, embolization, lower limb veins insufficiency, pelvic venous insufficiency, venous compartments, venous disease

## Abstract

Pelvic venous insufficiency is a common problem in multiparous women. Besides burdensome symptoms, it correlates with the development of venous disease in the lower limbs. Therefore, the sequential treatment of abdominal/pelvic before leg veins could improve treatment effectiveness. The medical records of 243 patients with venous disease who were subjected to sequential treatment were analyzed retrospectively. The symptoms and patient satisfaction were assessed using dedicated questionnaires, both before and after treatment. Clinical effectiveness was verified using a Doppler scan, both before and after treatment. Among 243 analyzed cases, 195 underwent whole treatment; however, 48 women after embolization did not require further intervention. The total-symptom-score change (11.6 vs. 13.0, respectively) and the satisfaction score (1.6 vs. 1.5, respectively) did not differ between groups. After embolization, some patients, besides symptoms improvement, experienced reflux reduction and, hence, might avoid further intervention. A better explanation for this beneficial effect of the sequential/descending approach requires further studies.

## 1. Introduction

Pelvic venous insufficiency (PVI) is a common health problem in multiparous women. Its main symptoms include chronic pain or discomfort in the hypogastric or pelvic region, menstrual-cycle abnormalities, predominantly excessive and/or prolonged menorrhea, dyspareunia and vulvodynia, dysuria, and varicosities in the vulvo-perineal region [1,2,3]. Moreover, in some women, the occurrence of PVI correlates with the development of lower-limb venous insufficiency (LLVI). Although some authors still consider the treatment in both compartments to be independent, denying any benefit from the PVI treatment in regard to LLVI [4], others suggest the rationale to treat PVI before the venous intervention in the lower-limb compartment [5,6]. One could expect that such a sequential or “descending” approach would allow the prevention of LLVI development or, presumably, its recurrence.

Currently, the introduction of less invasive techniques enabled the aforementioned treatment with the use of local anesthesia in one-day procedure settings, which reduced the risk of typical open-surgery-related complications and shortened recovery time [6,7,8]. According to the recent Guidelines of the International Union of Phlebology and the European Society for Vascular Surgery, the endovenous embolization of pelvic veins has become the first-choice method in PVI treatment [9,10]. Nowadays, various minimally invasive endovascular procedures are also preferred in LLVI treatment [10]. Thus, the increased safety with better clinical results, as compared to the surgical approach, could enable clinicians to perceive the real advantages of the complex endovascular treatment of venous insufficiency, based on “descending” intervention in adjacent compartments.

Although the abovementioned strategy follows recently published guidelines, it is still considered informal and raises a doubt with poor acceptance, especially among some vascular surgeons and more conservative phlebology experts [4]. In fact, the current experience is limited to relatively small patient groups, with the lack of sufficiently reliable data from larger studies or randomized trials [5,6]. Therefore, the aim of this study was to further extend this knowledge with a set of retrospective data concerning the short-term clinical outcome of the “descending” treatment of patients with venous disease who are suffering from both PVI and LLVI.

## 2. Materials and Methods

We performed a retrospective assessment of the data set extracted from the medical database of the clinic. It consisted of individual medical records that were collected during the routine diagnostic and treatment procedures, according to the optimized protocol of our clinic, as described in detail elsewhere [11].

Although the formal approval is not required for the retrospective assessment, the study protocol was submitted to the Local Ethics Committee at the Medical University of Warsaw (Statement No. AKBE/181/2020).

Briefly, all patients attending the clinic were routinely subjected to a detailed examination via a duplex-doppler ultrasound scan of their venous system of abdominal/pelvic compartment and lower limbs, using a Toshiba Xario 100 (TOSHIBA/Canon Medical Systems Co., Otawara, Tochigi, Japan) equipped with a convex (6–9 MHz) and a linear probe (8–14 MHz). Any detected or suspected abnormalities in abdominal or pelvic veins were further verified using either magnetic resonance venography (MRV) or computed tomography venography (CTV). The technical details of the MRV and CTV examinations were described previously [12].

Patients with confirmed diagnosis and with clinical symptoms of pelvic venous insufficiency were qualified for a routine treatment using minimally invasive endovenous embolization of affected pelvic veins [9,10]. Before the treatment, patients were asked to respond to a venous-disease-oriented online questionnaire focused on both abdominal/pelvic and leg symptoms [3,6,13,14]. The symptoms’ intensity was assessed using a 0–10 points visual analogue scale (VAS), with “0” corresponding to “no symptom present”, whereas “10” represented the “maximal symptom intensity”. The sum of scores for six main symptoms, including abdominal/pelvic and leg pain/discomfort, was shown as the total symptom score (TSS), with the min–max range from 0 to 60 points.

Pelvic veins’ embolization was performed as a day-case procedure with mild intravenous sedation, using the right trans-basilic and/or common femoral vein approach for venous cannulation and catheterization. The affected veins were occluded using pushable Nester^®^ (Cook Medical, Bloomington, IN, USA) and/or detachable Concerto^®^ (Medtronic/Micro Therapeutics Inc., Irvine, CA, USA) embolization coils, combined with foam sclerotherapy, under the supervision of a mobile C-arm X-ray/fluoroscopy device Zenition 70 (Philips Medical Systems, Best, The Netherlands). Approximately 4–6 weeks after embolization, patients underwent subsidiary ultrasound-guided foam sclerotherapy (UGFS) of persisting vulvo-perineal varices and/or pelvic–thigh shunts at “pelvic floor leakage points”. After the next 4–6 weeks, all women were subjected to a control ultrasound scan. Apart from the assessment of pelvic veins’ occlusion efficacy, the examination was focused on the presence/persistence of clinically significant (>0.5 s) venous reflux in the superficial veins of the lower-limb compartment. If the reflux was detected, patients were subjected to further treatment using laser thermoablation alone (for isolated truncal insufficiency) or in combination with sclerotherapy or phlebectomy (for incompetent tributaries). Patients without clinically relevant reflux were left without intervention and appointed for periodic control. Approximately 6 months after the last intervention, all patients were controlled using an ultrasound scan and were requested to respond to the second online survey, similar to that before treatment. In addition to the same assessment of symptoms’ intensity, patients were asked to evaluate their overall satisfaction with the treatment, using the following scale: “(1) fully satisfied (significant improvement)”, “(2) partially satisfied (moderate improvement)”, “(3) hesitant (weak or no improvement)”, and “(4) unsatisfied (worsening)”. The data collected during all procedures were successively stored in a password-protected database. For the easier comparison between groups or individuals within each group, the total symptom score change (TSSC) was calculated using the formula “TSSC = TSS before treatment—TSS after treatment”. The “0” value reflected no symptom change, the values “>0” corresponded to the improvement with symptoms intensity decrease, and “<0” reflected the symptoms having increased and worsened.

To exclude the sex-related data variability, the analysis was limited to females only. Thus, the primary search in database was performed using the following inclusion criteria: main diagnosis—PVI and LLVI; intervention—pelvic veins embolization; sex—female; age—adults (≥18); search range, 2019–2021; and available data from both before- and after-treatment surveys. The records extracted from the database were anonymized and further allocated into two subgroups, depending on the treatment being applied. Accordingly, records of patients with the significant reflux persisting in lower-limb veins after PV embolization and who underwent whole/sequential treatment were designated the “PV + LLV” group. The data of women without reflux in the LLV compartment after PV embolization and who were not subjected to further intervention were marked as belonging to the “PV” group. The data evaluation concerned selected clinical parameters and survey scores in both groups, both before and after treatment.

The basic statistical assessment included either the descriptive statistics or the Mann–Whitney U test, with a *p* < 0.05 being considered statistically significant.

## 3. Results

The database search resulted in the extraction of 243 records, which conformed to previously specified inclusion criteria. All patients revealed ultrasonographic and radiological features of PVI grade I/II or above, according to the simplified clinical classification described in detail elsewhere [15], as well as an LLVI of C2-C3 grade based on the updated clinical, etiological, anatomical, and pathological (CEAP) classification [16]. According to the baseline diagnostic findings, all patients were preliminarily qualified for three-step sequential/“descending” treatment: PV embolization first, then UGFS of pelvic–thigh shunts, and, finally, LLV-directed procedure.

The PV embolization concerned the left ovarian vein (LOV) in 96.7% of patients in the whole group, whereas the right ovarian vein (ROV) required intervention in 60.1% of cases. The embolization of the internal iliac vein or some collaterals was performed in 2.1% and 4.5% patients, respectively. The balloon venous angioplasty of the constricted left renal vein (LRV) was necessary in 20.2% of cases.

The assessment of data from the interim ultrasound control showed that the PV embolization with subsequent UGFS of pelvic–thigh shunts resulted in the effective occlusion of treated veins in all 243 patients. In 195 individuals (80.2% of the whole group), despite the considerable decrease in LLV overload, the reflux in the LLV compartment was still observed. These women were subjected to the further treatment, according to primary qualification (PV + LLV group). Unexpectedly, after embolization with UGFS, 48 women (19.7%) experienced a significant reduction in reflux in LLV, below the level of clinical relevance (<0.5 s). Hence, these patients, although preliminarily qualified for whole sequential treatment, including the LLV compartment, did not undergo any supplementary interventions in regard to the lower-limb veins (PV group).

The comparison of selected baseline parameters between women from both groups did not reveal any statistically significant difference in regard to their age, mean number of deliveries, percentage of postmenopausal, or the frequency of recurrent venous disease. However, despite similar mean number of pregnancies in both groups, the frequency of nulli- and primiparous women was statistically significantly lower in the PV group. Consequently, the occurrence of women experiencing two or more pregnancies in that group was higher than in the PV + LLV group. Noticeably, in patients from the PV group, the endometriosis was diagnosed approximately 3.6-fold more often than in those from the PV + LLV group.

The distribution of major abnormalities in abdominal/pelvic veins and the embolization procedure differed between groups. Approximately 25% of interventions in the PV group and 44% in the PV + LLV group were unilateral and concerned LOV only. In 56% of women from the PV + LLV group and 75% from the PV group, the embolization procedure involved both LOV and ROV. The balloon venous angioplasty of constricted LRV was necessary in 22.9% cases in the PV group and 19.5% in the PV + LLV groups, but this difference was non-significant. The mean score for pelvic pain/discomfort reported by patients before embolization was higher in the PV group when compared to the PV + LLV group, but this difference was non-significant. On the other hand, the mean score for leg pain/discomfort experienced by patients before the treatment was significantly higher in the PV + LLV group, compared to the PV.

Nearly one-third of patients from the whole group underwent some venous interventions in the past. Their occurrence ranged from 25% in the PV group up to 37.9% in the PV + LLV group, and this difference was statistically significant. Notably, 86% of patients with recurrent venous disease after embolization still required further treatment in the LLV compartment. The odds for the necessity of some auxiliary procedures in patients with a history of previous venous treatment were slightly higher, as compared to intact patients, with the odds ratio (OR) being 1.83 and the 95% confidence interval (CI) being 0.90–3.75, but with a low significance level of *p* =0.096.

The baseline clinical data of the whole group, as well as the comparison of selected baseline features between the PV and PV + LLV subgroups, are shortly summarized in Table 1.

The mean total symptom score before treatment was similar in both groups. After the treatment, the mean TSS decreased significantly, but it did not differ between groups. Hence, despite the slight difference in the mean value of the total-symptom-score change (TSSC) between groups, it did not reach statistical significance either.

**Figure 1:**
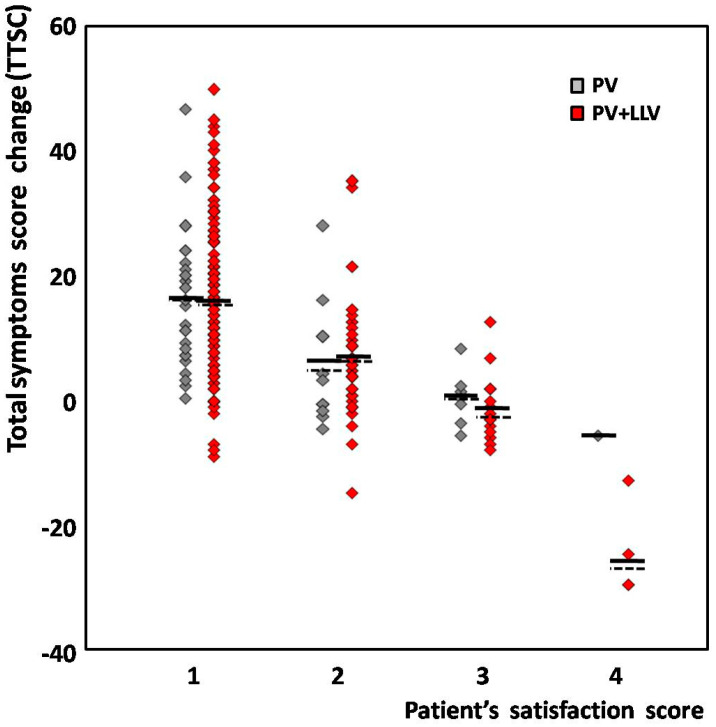
The distribution of total symptom score change (TSSC) in regard to patient’s satisfaction score. PV (gray)—patients subjected to pelvic veins embolization only; PV + LLV (red)—patients subjected to whole/sequential treatment (including lower limb veins). Each diamond represents one patient; horizontal solid and dashed lines correspond to the mean and median for each group, respectively.

When analyzing the difference between groups in regard to the patients’ satisfaction score, the mean TSSC appeared similar, except from that of unsatisfied patients from the PV + LLV subgroup, who reported more pronounced worsening compared to one PV patient. However, this difference did not reach statistical significance (Figure 1).

The assessment of satisfaction scores’ distribution between groups showed the higher prevalence of significant improvement in PV + LLV, whereas the occurrence of “weak or no improvement” scores in the same group was lower, compared to the PV group. The frequency of other satisfaction scores did not differ among groups (Table 2).

After the entire treatment, at least a moderate improvement was reported by 214 patients (88.1%) from the whole group; however, in 207 women (85.2%), the calculated TSSC was >0. In fact, although the individual TSSC and the patient’s satisfaction score revealed a significant correlation (with R= −0.58; at *p* = 0.00001), in 11.9% of cases, some discrepancy between both variables was observed. Among 159 satisfied women from the whole group who declared significant improvement, in 5 patients (3.1%), at the same time, the mean TSSC was −5.4 ± 3.6, which may suggest slight worsening. All of these individuals were from the PV + LLV group. Moreover, 10 of 55 patients (18.2%) with moderate improvement had a TSSC < 0. The distribution of individual TSSCs in regard to the patient’s satisfaction score for both groups was shown on Figure 1.

One woman from the PV group who was unsatisfied with treatment results reported an increase in pelvic symptoms after embolization. However, in this case, the endometriosis was also diagnosed. Four women from the PV + LLV group who were unsatisfied with treatment results reported marked worsening in their post-treatment surveys (mean TSSC −24.5 ± 8.0), mainly in regard to leg symptoms. Surprisingly, their control ultrasound scan, which was performed after the entire treatment, did not reveal any clinically relevant reflux in superficial veins. According to available visit reports, the only abnormalities remaining in the LLV compartment were small reticular veins and telangiectasiae, located on the postero-lateral aspect of the thigh, which apparently affected the overall patient’s satisfaction. The summary of particular symptom scores in the assessed groups, both before and after treatment, was shown in Table 2.

## 4. Discussion

Regardless of the increasing knowledge concerning the pathophysiology of the pelvic venous system, its clinical relevance is still underappreciated. Regrettably, although the CEAP 2020 revision and recent ESVS Guidelines highlighted the involvement of PVI in the development and treatment of venous disease [10,16], so far, the awareness of their role remains unsatisfactory among clinicians.

As shown in our recent study, undiagnosed PVI seems to be the main cause of recurrence after previous LLV treatment [12]. Hence, the “descending”/sequential treatment (PV first and LLV next) should better correct the origin of hemodynamic abnormalities and improve the effectiveness of the entire treatment. Nevertheless, despite some attempts to standardize this subject [17,18], the lack of a common standard in diagnostics and the implementation of various treatment approaches in PVI studies make the reliable comparison of results from those studies difficult or even impossible [2,6]. Presumably, the diverse interpretation of venous system anatomy or, more likely, the misunderstanding of its hemodynamic implications could be an issue. One such example could be the connections between the pelvic and thigh veins. Besides the ilio-femoral axis, the so-called pelvic “leakage points”, as well as the vulvo-perineal veins, seem to be even more crucial. While negligible in normal conditions, in some patients with PVI, they expand and form a large blood reservoir of the “pelvic floor” compartment. It is noteworthy that the direct management of that compartment is usually not included in the routine embolization procedure [3,7,9]. On the other hand, the renouncement of its reduction might attenuate the benefit from PV embolization and diminish its hemodynamic impact on the LLV compartment, as well as the long-term outcome [6].

This hypothesis could be supported by the observation from our study, concerning nearly one-fifth of assessed cases. Although preliminarily qualified for the sequential intervention in PV and LLV compartments, after the embolization followed by UGFS of the “pelvic floor” compartment, they experienced significant improvement, which allowed at least the temporary postponement of LLV procedures and spared the main venous trunks. The aforementioned observation, besides directly justifying the benefit from the sequential hemodynamic management of venous disease, also confirms the key role of pelvic veins in the pathogenesis of LLV insufficiency [5].

Apart from hemodynamic improvement, as recorded in the impartial ultrasound assessment, the important, although more subjective, indicator of treatment efficacy could be the symptoms’ reduction [1,6,13]. It is worth noting that a vast majority of patients experienced at least moderate improvement, with a significant reduction intheir total symptom score. Only a few cases reported no improvement or even worsening. The latter, however, was supposed to be contributed by unrelated circumstances, such as endometriosis or some persisting angiectasiae without clinical relevance.

The factors influencing the treatment efficacy overlap with those involved in the pathogenesis of venous disease in general and PVI in particular. Besides various anatomic abnormalities in the venous system, the main variables affecting the treatment outcome appear to be the history of pregnancy and previous interventions in the LLV compartment [12]. According to our previous studies, women experiencing three or more term pregnancies/deliveries are more prone to develop advanced venous disease [11]. Moreover, they are more likely to have already undergone some LLV intervention in the past. It is noteworthy that patients with recurrent venous disease required LLV treatment after PV embolization more often compared to others. Especially in those patients, such “descending”/sequential treatment, although being technically more difficult, mainly due to various post-intervention alterations in the venous system (e.g., overload of deep veins), resulted in better improvement, either in regard to symptoms’ reduction or overall patient satisfaction.

It should be emphasized that the aforementioned observation is further supported by our over-8-year experience with “descending”/sequential treatment, with only a few unsuccessful cases. Those cases include patients with limited possibility to perform the fully effective embolization, e.g., with highly developed collateral circulation, or severe alterations in venous anatomy. The latter concern subjects after previous negligent surgery in the LLV compartment, especially those with non-hemodynamic high ligation and stripping of great saphenous vein. Therefore, to avoid the recurrence, both the detection and elimination of PVI, prior to the primary intervention in the LLV compartment, seem to be of great importance.

The main limitation of our study is its retrospective nature. Hence, due to some missing data, we could not include several important parameters, e.g., the diameters of lower-limb veins before and after embolization. Instead of that, we used the results of the reflux assessment, with a threshold level =0.5 s for its clinical relevance. It is worth noting that, in the vast majority of patients, this approach was sufficient to observe the decrease in LLV overload after pelvic veins’ treatment. Nevertheless, to better demonstrate the hemodynamic impact of the embolization procedure on the LLV compartment, the veins’ diameters should also be assessed in further research, besides their competence.

The second issue to be emphasized is the specific selection of studied cases. Definitely, our results should not be extrapolated to the whole population. While all patients analyzed in our report had been diagnosed with PVI, obviously it is not the case for all patients with venous disease. This awareness particularly applies to statistics of reflux patterns, as approx. 70% of our patients presented a fully competent terminal valve.

In conclusion, our report provides some additional data to improve our understanding of the pathophysiology of venous disease and factors which may determine the outcome of its treatment. With more than 80% of satisfied patients, our data support the rationale to use the sequential/“descending” protocol in the management of women with venous disease. However, a better explanation of the beneficial effect of this approach still requires further multicenter studies.

## Figures and Tables

**Table 1:** Clinical characteristics of the groups.

Feature/Parameter	Whole Group (*n* =243)	PV (*n* =48)	PV + LLV (*n* =195)
Age (mean ± SD; median)	41.5 ± 9.1; 40.0	41.7 ± 10.9; 40.0	41.4 ± 8.6; 40.0
Number of pregnancies	2.0 ± 1.2; 2.0	2.3 ± 1.1; 2.0	2.0 ± 1.2; 2.0
(mean ± SD; median)			
0 (%)	14.0	8.3	15.4
1 (%	14.0	4.2	16.4
2 (%)	41.6	54.2	38.5
3+ (%)	30.4	33.3	29.7
Number of deliveries (mean ± SD; median)	1.8 ± 1.1; 2.0	2.0 ± 1.0; 2.0	1.7 ± 1.1; 2.0
Postmenopausal (%)	13.1	10.4	13.8
Leg pain at standing/sitting (VAS 0–10) (mean ± SD)	4.9 ± 2.8	4.3 ± 2.5	5.1 ± 2.8
Previous leg treatment (%)	35.4	25.0	37.9
Pelvic pain/discomfort (VAS 0–10)(mean ± SD)	3.9 ± 3.3	4.2 ± 3.1	3.8 ± 3.3
Endometriosis confirmed (%)	7.0	16.7	4.6
Pelvic veins affected (%):			
LOV	96.7	100.0	95.9
ROV	60.1	75.0	56.4
Collaterals	4.5	6.2	4.1
IILV	2.1	2.1	2.1
LRV constriction (%)	20.2	22.9	19.5

Abbreviations: PV—“pelvic veins embolization only” group; PV + LLV—“pelvic and lower limb veins sequential treatment” group; VAS—visual analogue scale; LOV—left ovarian vein; ROV—right ovarian vein; IILV—internal iliac veins; LRV—left renal vein.

**Table 2:** Clinical symptoms and their changes following the treatment.

Feature/Parameter	Whole Group *(n* = 243)	PV (*n* = 48)	PV + LLV (*n* = 195)
Total symptoms score (TSS): - before treatment - after treatment (Mean ± SD; median)	23.2 ± 14.5; 23.0 10.5 ± 9.0; 8.0	22.6 ± 13.0; 22.5 11.0 ± 9.8; 8.0	23.4 ± 14.9; 23.0 10.4 ± 8.9; 8.0
Total symptoms score change (TSSC): (Mean ± SD; median):	12.8 ± 13.6; 11.0	11.6 ± 11.7; 9.5	13.0 ± 14.0; 12.0
Leg pain at standing position: - before treatment - after treatment (VAS 0-10: Mean ± SD; median)	4.9 ± 2.8; 5.0 2.3 ± 2.1; 2.0	4.3 ± 2.5; 4.0 2.5 ± 2.3; 2.0	5.1 ± 2.8; 5.0 2.3 ± 2.1; 2.0
Pelvic pain (menorrhea-unrelated): - before treatment - after treatment (VAS 0-10: Mean ± SD; median)	3.9 ± 3.3; 4.0 1.7 ± 2.2; 1.0	4.2 ± 3.1; 5.0 1.8 ± 2.0; 1.0	3.8 ± 3.3; 3.0 1.7 ± 2.2; 1.0
Leg pain during menorrhea: - before treatment - after treatment (VAS 0-10: Mean ± SD; median) (* Excluding postmenopausal cases)	4.8 ± 3.2; 5.0 (* 5.1 ± 3.1; 5.0) 2.3 ± 2.4; 1.0 (* 2.5 ± 2.4; 2.0) (* *n* = 210)	4.2 ± 3.0; 4.0 (* 4.8 ± 3.0; 4.5) 2.2 ± 2.5; 1.0 (* 2.6 ± 2.6; 2.0) (* *n* = 42)	4.9 ± 3.3; 5.0 (* 5.2 ± 3.2; 5.0) 2.3 ± 2.4; 1.0 (* 2.5 ± 2.4; 2.0) (* *n* = 168)
Dyspareunia: - before treatment - after treatment (VAS 0-10: Mean ± SD; median)	3.0 ± 3.1; 2.0 1.2 ± 1.9; 0.0	2.9 ± 3.2; 2.0 1.3 ± 2.2; 0.0	3.0 ± 3.1; 2.0 1.2 ± 1.8; 0.0
Pelvic pain at exercise/load: - before treatment - after treatment (VAS 0-10: Mean ± SD; median)	3.1 ± 3.0; 2.0 1.3 ± 1.8; 1.0	3.2 ± 3.0; 2.0 1.6 ± 2.3; 1.0	3.0 ± 3.0; 2.0 1.3 ± 1.7; 0.0
Self-assessment of venous disease: - before treatment - after treatment (VAS 0-10: Mean ±SD; median)	6.5 ± 2.4; 7.0 2.8 ± 2.1; 2.0	6.4 ± 2.6; 7.0 3.3 ± 2.0; 3.5	6.5 ± 2.4; 7.0 2.6 ± 2.1; 2.0
Distribution (number and %) of satisfaction score: (1) fully satisfied (significant improvement) (2) partially satisfied (moderate improvement) (3) hesitant (weak or no improvement) (4) unsatisfied (worsening)	159 (65.4%) 55 (22.6%) 24 (9.9%) 5 (2.1%)	28 (58.3%) 11 (22.9%) 8 (16.7%) 1 (2.1%)	131 (67.2%) 44 (22.6%) 16 (8.2%) 4 (2.1%)
Patient’ satisfaction score: (VAS 1-4: Mean ± SD; median)	1.5 ± 0.8; 1.0	1.6 ± 0.8; 1.0	1.5 ± 0.7; 1.0

Abbreviations: PV—“pelvic veins embolization only” group; PV + LLV—“pelvic and lower limb veins sequential treatment” group; VAS—visual analogue scale.

## Data Availability

Due to restrictions aimed to protect patient confidentiality, the original data are publicly not available; however, the data presented in this report are available from the corresponding author upon request.
